# Seed coat thickness in the evolution of angiosperms

**DOI:** 10.1007/s00018-018-2816-x

**Published:** 2018-05-05

**Authors:** Olivier Coen, Enrico Magnani

**Affiliations:** 1 Institut Jean-Pierre Bourgin, INRA, AgroParisTech, CNRS, University of Paris-Saclay, Route de St-Cyr (RD10), 78026 Versailles Cedex, France; 2 Ecole Doctorale 567 Sciences du Végétal, University Paris-Sud, University of Paris-Saclay, bat 360, 91405 Orsay Cedex, France

**Keywords:** Seed coat, Integument, Fertilization, Ovule, Seed evolution, Seed maternal tissues, Seed coat thickness

## Abstract

The seed habit represents a remarkable evolutionary advance in plant sexual reproduction. Since the Paleozoic, seeds carry a seed coat that protects, nourishes and facilitates the dispersal of the fertilization product(s). The seed coat architecture evolved to adapt to different environments and reproductive strategies in part by modifying its thickness. Here, we review the great natural diversity observed in seed coat thickness among angiosperms and its molecular regulation in Arabidopsis.

## Introduction

The plant sexual reproduction cycle—meiosis, sex differentiation, and fertilization—times the alternation of haploid and diploid generations [1]. Mitosis intercalates the cycle phases and determines the predominance of one generation versus the other. Whereas most non-vascular plants display a predominant gametophytic phase, vascular plants tend to develop large sporophytes [1]. Non-seed plants develop gametophytes physically separated from the sporophyte through the dispersal of spores. By contrast, the evolution of the seed habit in gymnosperms and angiosperms marked the retention of the female gametophyte on the sporophyte and the dispersal of the zygotic embryo (next sporophytic generation) [63]. Seeds greatly contributed to the successful colonization of land by vascular plants. Compared to spores, seeds carry nutrients, rely less on water for germination, convey a higher degree of protection to physical stress, and can disperse in different ways. Such evolutionary advantages were, to a great degree, achieved through the evolution of the seed coat, cell layers that surround, protect, and facilitate the dispersal of the seed fertilization product(s) [52].

**Fig. 1:**
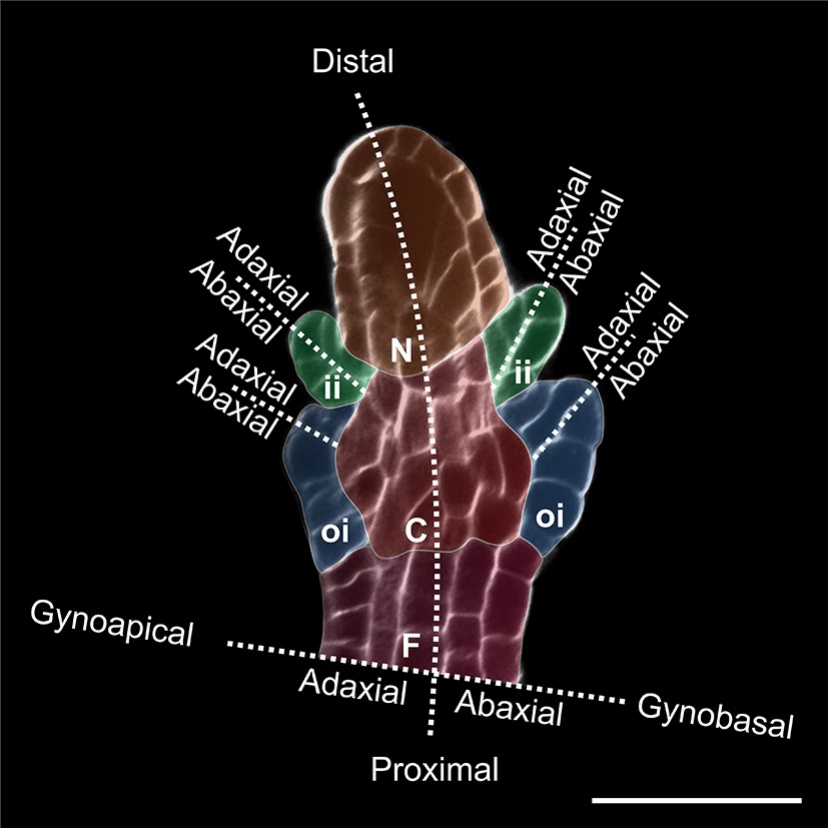
Arabidopsis ovule primordium at stage 2-V stained with Calcofluor M2R White. *F* funiculus (violet), *C* chalaza (red), *N* nucellus (orange), *oi* outer integument (blue), *ii* inner integument (green). Bar = 20 µm

The seed coat is a maternal sporophytic tissue that originates from the ovule integument(s). A typical ovule (the seed precursor) comprises four sporophytic tissues: (1) the funiculus, which transports nutrients from the placental tissue, (2) the chalaza, which forms one or more protective (3) integuments, and (4) the nucellus, whose megaspore mother cell undergoes meiosis to originate the gametophyte (Fig. 1). Ovule integuments grow as primordia from the chalaza and are referred to as dermal, if initiated solely by chalazal dermal cells, or sub-dermal, if originated by chalazal dermal and sub-dermal tissue [18, 43]. The developmental patterning of integuments has been thoroughly analyzed in *Arabidopsis thaliana* [13, 24, 72]. Arabidopsis plants display an outer (oi) and an inner (ii) integument that develop from the chalaza of a finger-like protruding ovule primordium (Fig. 1). The ii primordium initiates as a ring-like structure from dermal chalazal cells (ii initials) at the boundary with the nucellus. ii initials become visible when they undergo a periclinal or oblique cell division followed by cell elongation. The oi primordium grows adjacent to the ii proximal side and extends to the proximal extremity of the chalaza. Whereas the distal part of the oi primordium is of dermal origin and follows ii ontogeny, the basal part is subtended by sub-dermal chalazal tissue undergoing periclinal cell divisions. ii and oi annular primordia grow by anticlinal cell divisions to surround and shape the nucellus and female gametophyte. The distal extremity of the integuments forms the micropyle, a small opening through which the pollen tube penetrates. After fertilization, integument cell layers follow different pathways of cell differentiation [34] and grow in coordination with the fertilization product(s) [40]. Finally, the seed coat undergoes programmed cell death and establishes a protective barrier in between fertilization product(s) and surrounding environment [41].

Seed coat structure and molecular composition deeply influence seed physiology and evolved to adapt to different environmental conditions [43]. Natural diversity has been observed in the thickness of the seed coat, which affects seed germination, dormancy, and mortality [27, 47, 60]. Overall, seed coat thickness is determined by both integument number and cell thickness. Such a character changes along the seed polarity axes and varies among individuals and species. This review explores the evolutionary patterns of seed coat thickness development in angiosperms.

## Natural diversity in seed coat thickness

### Integument number

**Fig. 2:**
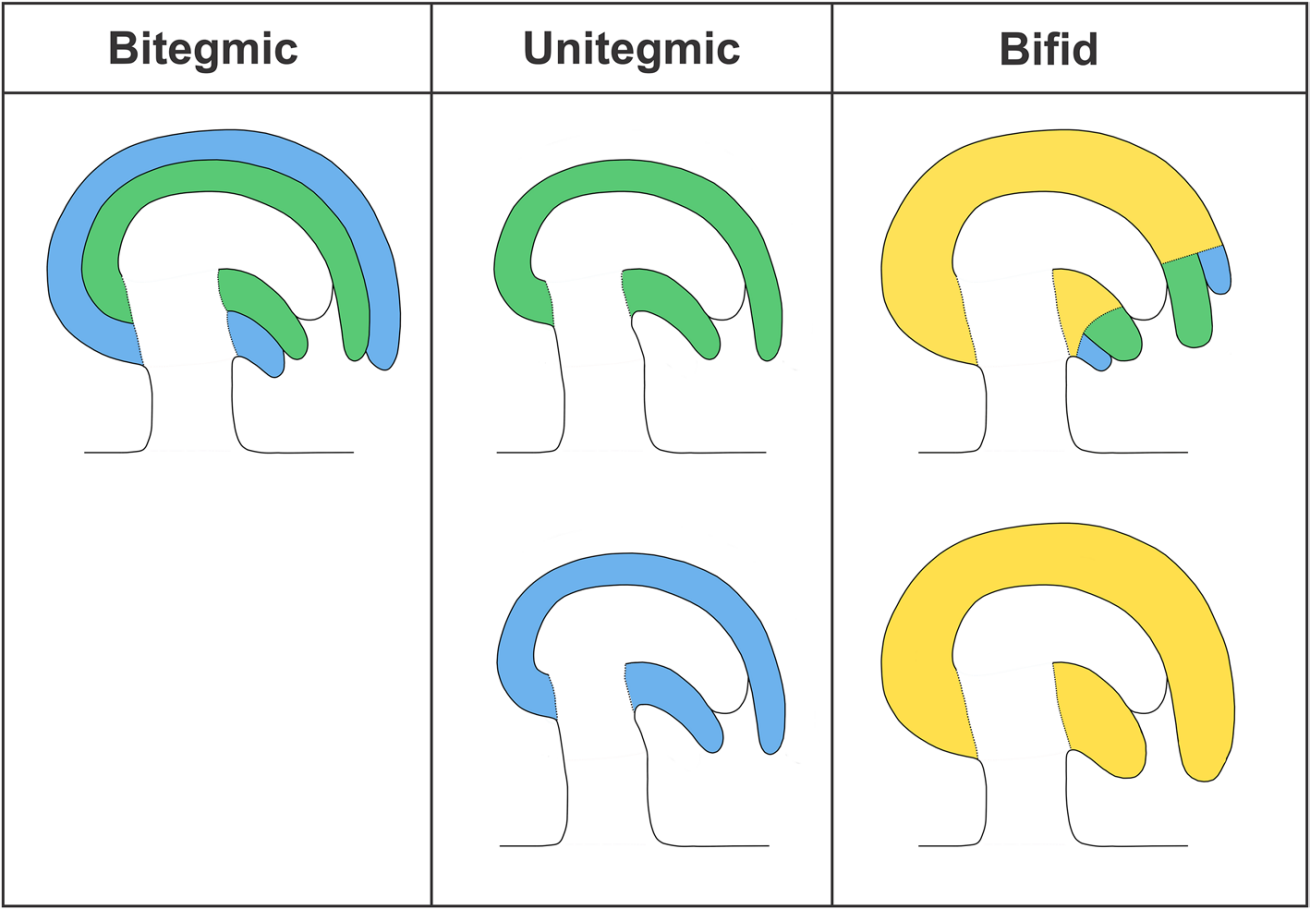
Scheme of bitegmic, unitegmic, and bifid ovules. Outer integument (blue); inner integument (green); tissue of unclear origin (yellow)

Fossil records of Paleozoic pre-ovules from the earliest seed plants revealed the presence of only one integument (unitegmic, Fig. 2) [28, 80]. The integument structure of such pre-ovules varied among species, but it was typically lobed, vascular bundled, and detached from the nucellus. Leaf-like structures, termed cupules, have been found to partially enclose the ovule and are commonly viewed by paleo-botanists as the precursor of a second integument. Extant gymnosperm ovules are also unitegmic and the integument is probably homologous to that of ancestral seed plants. By contrast, angiosperms evolved two integuments (bitegmic), the so-called oi and ii (Fig. 2). Phylogenetic analyses suggest that bitegmy is the ancestral condition in angiosperms, nevertheless unitegmy arose several times during angiosperm evolution [18].

The transition from bitegmic to unitegmic ovules happened several times during angiosperms evolution along two major ontogenic pathways: (1) developmental suppression or retardation of one integument, (2) congenital integument fusion. Morphological analyses in the *Piperaceae* family revealed that the single integument of *Peperomia* shows similarity to the ii of *Piper*, suggesting that the oi of the former has been lost [88]. Similarly, early arrest of ii has been described in *Populus* [81]. Congenital fusion of ii and oi probably arose through different mechanisms. Genetic analyses in Arabidopsis (see below) suggest that integumentary fusion can be achieved through lack of boundary formation between oi and ii [14, 44]. This interpretation is confirmed by the analysis of molecular markers of boundary formation in *Prunus* bitegmic and unitegmic ovules [53] and by morphological studies in *Anacardiaceae* and *Balsaminaceae* [43]. By contrast, a mechanism of integumentary shifting has been proposed for the development of the single integument of a number of *Ranunculaceae* [6]. According to Bouman and Calis, oi sub-dermal tissue subtending ii initials undergoes periclinal cell divisions and, in conjunction with oi, shifts the ii upward. An incision at the tip of the single integument reveals oi and ii remnants. According to the authors, this mechanism would create a bifid integument in between uni- and bitegmic and would only be possible in ovules carrying a sub-dermal oi and dermal ii (Fig. 2). The process of integumentary shifting has been also invoked to explain the development of the unitegmic *Gentiana* ovule, where no oi and ii remnants are visible [7] (Fig. 2). According to this model the sequence and position of dermal and sub-dermal cell divisions might have led to a unitegmic ovule through integumentary shifting of a sub-dermal oi and dermal ii. Interestingly, species with bitegmic, bifid and unitegmic ovules are grouped in the *Impatiens* gender. Boesewinkel and Bouman have presented the development and evolution of *Impatiens* bifid and unitegmic ovules as integumentary shifting [5]. More recently, McAbee et al. have revised this theory and described a change of sub-dermal growth from beneath the oi to the region beneath both oi and ii, without invoking an upward shift of the ii [55]. According to this hypothesis, unitegmy would be the result of ii–oi intercalary growth. All considered, we believe that available data on integument congenital fusion in angiosperms point to two non-exclusive mechanisms: (1) total or partial lack of boundary formation and (2) intercalary growth between ii and oi primordia, regardless of their dermal or sub-dermal origin.

Further ovule reduction has been observed in *Santalales* [74]. Members of this clade do not develop any ovule integument (ategmy). Expression analyses revealed that integument molecular markers are expressed at the distal end of *Santalales* ategmic ovules [10]. These data have been interpreted as the fusion of an integument remnant to the nucellus. By contrast, some angiosperms evolved a third cell sheath in addition to oi and ii. *Annonaceae* ovules develop a so-called third integument in between oi and ii [12], whereas Myristicaceae and other groups initiate a third envelop, referred to as aril (not to be confused with arillodes), later on in development in a region proximal to the oi that has been interpreted as the funiculus [17, 66, 77]. Interestingly, arils can also be found in gymnosperms, such as *Taxus baccata* [54]. Arils either work as attractive organs that facilitate seed dispersal or are reduced to a tiny protuberance, as in a number of basal angiosperms [39].

Finally, the number of integument cell layers can change after ovule fertilization during early seed development. Rice ovules display two-cell-layered oi and ii. Within 3 days after fertilization, oi and ii of most rice cultivars are absorbed by the pericarp leaving only the cuticle layer [50].

### Integument cell thickness

Two-cell-layered dermal integuments originate from the chalazal dermal tissue, whereas three-cell-layered sub-dermal integuments display two dermal and one sub-dermal cell layer. Furthermore, integuments can develop more cell layers through periclinal cell division of sub-dermal or dermal cell layers and are referred to as multiplicative [43]. Integument cell thickness is considered a stable character worth using in macrosystematic analyses [18]. Nevertheless, 3D morphological analyses of Arabidopsis ovules suggest the contrary. Arabidopsis ovules were thought to display a regular two- and three-cell-layered oi and ii, respectively [72]. The ii grows a third cell layer (ii1′) by periclinal cell divisions of the innermost ii cell layer (ii1). Coen et al. have described the formation of a fourth cell layer (ii1″) by periclinal cell division of the ii1′ in 70 and 35% of *Wassilewskija* and *Columbia* Arabidopsis ovules, respectively [13]. Furthermore, the oi has been recently shown to initiate sub-dermal cell stripes (oi′) in 10% of the *Columbia* Arabidopsis ovules tested [24]. These data highlight the importance of 3D imaging for the analysis of integument cell patterning and suggest that cell thickness is a less stable character than previously thought.

Finally, integument thickness is affected by cell size. During ovule development, cell size does not vary considerably among integument cell layers [72]. Nevertheless, integument cell layers follow different fates and expand dishomogeneously after fertilization. In Arabidopsis, ii1′ and ii1″ undergo a more dramatic cell expansion process compared to the other integument cell layers. Coen et al. have revealed that ii1′ and ii1″ growth is not tightly coordinated with the rest of the integument cell layers and have suggested a role in cushioning seed coat development and offsetting perturbations in its developmental program [13].

### Integument thickness along the ovule polarity axes

Both integument number and cell thickness can vary considerably along the ovule polarity axes (Fig. 1). Integument growth leads to the formation of a micropyle at the distal side of the ovule. The majority of bitegmic ovules are amphistomic, as both oi and ii contribute to micropyle formation. More rarely, angiosperm ovules can be endostomic or exostomic if only the ii or the oi, respectively, generate the micropyle [18]. Endostomic ovules show distal thickening of the ii at the micropyle where the ii is not covered by the oi, suggesting that oi limits ii growth [38]. Thickening of the distal region of the integument might be instrumental to enclose the nucellus apex and form a micropylar pore. Proximal ii thickening is instead visible in Arabidopsis where ii1′ and ii1″ do not develop all the way to the micropyle. Ectopic ii1′ growth in the micropylar region interferes with embryo development by limiting its growth space and is repressed by a tight regulatory mechanism (see below) [13].

In several basal angiosperms, oi and/or ii radial patterning shows discontinuous growth and leads to lobation. Lobed iis have been considered remnants of ancestral integuments or a sign of atavism as paleozoic ovules displayed a lobbed integument [83]. Nevertheless, it has been argued that lobation might have evolved as a way to restrict the circumference of the integument and allow the formation of a micropyle, as an alternative mechanism to distal integument thickening [38].

Finally, integument number and cell thickness deeply affect ovule curvature along the adaxial–abaxial polarity axis. The micropyle of anatropous (curved) ovules is placed side by side to the chalaza, whereas orthotropous (non-curved) ovules develop micropyle and chalaza along a straight line. Orthotropous ovules are either unitegmic or display a thin or partially developed oi suggesting that the oi plays a major role in ovule curvature [18]. This hypothesis is supported by a number of Arabidopsis mutants that revert from anatropous to orthotropous when the oi is missing or partially developed (see below) [14, 44]. Nevertheless, gymnosperm *Podocarpaceae* develop unitegmic anatropous ovules. In such ovules, the function of the oi is taken over by the epimatium, an ovuliferous scale that resembles the oi and is involved in ovule curvature [82]. Angiosperm anatropous ovules have either annular or semi-annular oi primordia that grow into cup-shaped or hooded ois [92]. Ovules with a hooded oi display a difference in thickness along the ovule adaxial–abaxial polarity axis: the abaxial side shows a fully developed oi whereas the adaxial side misses partially or entirely the oi. The development of a hooded or cup-shaped oi morphology has been correlated to the speed of developmental curvature: pronounced semi-annular oi primordia tend to curve faster than annular primordia [18].

### Molecular regulation of seed coat thickness in arabidopsis

#### Establishment of the ovule proximal–distal polarity axis

In Arabidopsis, the ovule proximal–distal polarity axis is morphologically evident at stage 2 of ovule development when the distal nucellus develops a megaspore mother cell, the central chalaza initiates integument primordia, and the proximal funiculus differentiates a vascular strand (Fig. 1) [72]. Genetic and molecular evidences suggest that ovule proximal–distal patterning occurs sequentially starting from the distal nucellus toward the funiculus [73]. The establishment of chalazal identity, which has obvious repercussions on integument initiation, is therefore deeply influenced by nucellus development. More than 25 years of genetic studies on Arabidopsis ovule development have revealed a handful of genes involved in ovule proximal–distal patterning.

Mutations in the *AINTEGUMENTA* (*ANT*) gene, encoding for an APETALA2 domain transcription factor, lead to bare ovules with no or reduced integument primordia [16, 49]. Starting at stage 2, *ANT* is expressed in the chalaza and integuments primordia where it is speculated to regulate cell proliferation underlying integument outgrowth [16, 49]. Furthermore, ANT and the mitochondrial ribosomal protein HUELLENLOS (HLL) have redundant functions in establishing the ovule proximal–distal axis [73, 79]. Ipomorph *hll* alleles display early arrest of integument development and, in combination with *ant* mutations, lead to reduced chalaza and funiculus regions [73]. Similarly, HLL works synergistically with the SHORT INTEGUMENTS 2 (SIN2) mitochondrial DAR GTPase [9, 36]. *sin2* ovules show reduced integument growth, whereas *sin2;hll* double mutants undergo early arrest of ovule primordia [9]. The nature of HLL and SIN2 protein indicates that metabolic defects can affect developmental patterning through mechanisms yet to be discovered.

Another early marker of the chalazal region, the BEL1 homeodomain transcription factor, has been implicated in the acquisition of chalazal identity [59, 67, 69]. *bel1* ovules fail to develop an ii and the oi turns into a swollen collar-like tissue or secondary ectopic carpeloid structure, depending on the *bel1* mutation. A similar phenotype has been observed in ovules over-expressing the *AGAMOUS (AG)* MADS-box gene [67] and in the triple MADS-Box genes mutant *seedstick;shatterproof1;shatterproof2* (*stk;shp1;shp2*) [65]. Interestingly, the carpeloid outgrowths of *bel1* ovules are converted into green leaf-like structures in the double *apetala2;bel1* and quadruple *stk;shp1;shp2;bel1* mutants [8, 59]. Brambilla and coworkers have proposed a model for cell identity acquisition in ovule integuments based on genetic analyses and in vitro MADS-box proteins interactions [8]. According to it, BEL1 interacts in the chalaza with AG and SEPALLATA3 MADS-box proteins to repress carpel identity in the integuments; this protein complex is then stabilized by an ovule identity complex comprising STK, SHP1, and SHP2 proteins [8].

Whereas BEL1 and ANT work in a cell-autonomous fashion, the WUSCHEL (WUS) homeodomain transcription factor promotes integument initiation from the nucellus, non-cell autonomously [33]. *wus* ovules fail to develop integuments and show defects in megagametogenesis. *WUS* and *ANT* do not regulate each other’s expression suggesting that they establish independent pathways from the nucellus and chalaza, respectively. These data indicate that ANT determines responsiveness of chalazal cells to a WUS-mediated signal from the nucellus that ultimately induces integument formation. *WUS* expression in the nucellus is tightly regulated by a plethora of transcription factors. Defects in integument development of *bel1*, *sporocyteless/nozzle* (*spl/nzz*, see below), *stk;shp1;shp2*, and *phabulosa;phavoluta;corona* (*phb;phv;cna*, see below) mutants have been linked to ectopic *WUS* mRNA accumulation in the chalaza [8, 75, 93]. Furthermore, exogenous application of cytokinin on ovules leads to integument arrest and concomitant expression of *WUS* in the integuments [4]. Finally, the expression of *WUS* in the chalaza under the control of the *ANT* promoter region expands the chalazal domain and leads to the initiation of an ii and two or more proximal integuments, some of which express oi markers [33, 75]. Only the ii of *proANT:WUS* ovules grows around the nucellus, whereas more proximal integuments fail to develop further.

The adaptor-like transcriptional repressor SPL/NZZ, another master regulator of the ovule proximal–distal polarity axis, is expressed throughout the ovule at stage 2, but more strongly in the dermal cells of the chalaza and in integument primordia [2, 71, 90, 94]. *spl/nzz* ovules display reduced nucellus and integuments growth as well as impaired sporogenesis, a phenotype that is partially suppressed by the *bel1* mutation [2, 71, 94]. SPL/NZZ negatively regulates the expression of *ANT* and *BEL1* in the nucellus while positively regulating *WUS* expression [2, 75]. By contrast, *SPL/NZZ* expression in the chalaza is activated by ANT and BEL1 and it is necessary for the establishment of the adaxial–abaxial polarity axis, thus working as a link between proximal–distal and adaxial–abaxial patterning [2, 3].

#### Establishment of ovule and integuments adaxial–abaxial polarity axes

In Arabidopsis, ovule primordia acquire adaxial–abaxial polarity and transition from radial to bilateral symmetry at stage 2-III (Fig. 1) [72]. Whereas the ii primordium arises simultaneously on the adaxial and abaxial side of the ovule primordium, the oi primordium initiates on the abaxial face and its predominant abaxial growth is responsible for the Arabidopsis ovule curvature [18]. Adaxial–abaxial patterning characterizes then the laminar growth of each integument (Fig. 1). Here again, genetic studies have helped better understand regulatory pathways underlying such processes.

KANADI (KAN) and YABBY (YAB) transcription factors are known to promote abaxial identity in vegetative organs and ovules [48, 76]. *kan1;kan2* mutant ovules display reduced oi growth [21]. Similarly, mutations in the *YAB* gene *INNER NO OUTER (INO)* lead to the development of an amorphous protuberance at the place of the oi [85]. *INO* expression in the proximal–abaxial side of the chalaza (stage 2-I) is tightly regulated in a spatial–temporal fashion. Whereas BEL1 is a pre-requisite for *INO* expression, ANT and SPL/NZZ act antagonistically to time the onset of *INO* expression which in turn establishes a positive auto-regulatory loop and feedbacks on *ANT* expression [3, 58]. The SUPERMAN (SUP) zinc-finger protein interferes with INO positive regulatory loop in the adaxial domain of ovule primordia, promoting oi abaxial development and ovule bilateral symmetry [26, 58, 85]. Similarly, *WUS, SPL/NZZ, HLL,* and *KAN4/ABERANT TESTA SHAPE (ATS)* work redundantly in delimitating *INO* expression to the abaxial side of the oi [3, 75, 85]. Finally, INO has been shown to directly interact with the co-repressors LEUNIG and SEUSS and the co-activator ADA2b/PROPORZ1 (PRZ1) to promote oi growth [78]. Acquisition of ovule adaxial identity is instead promoted by the class III homeodomain-leucine zipper transcription factor PHB. *PHB* mRNA initially localizes in the adaxial side of ovule primordia (stage 1-I) [75] and *phb*-*1d/PHB* gain-of-function mutants, insensitive to miRNA negative regulation, exhibit orthotropous ovules with reduced oi growth [45, 57].

Abaxial–adaxial polarity appears also within integuments, with both oi and ii displaying an adaxial and abaxial side, necessary to establish the oi–ii boundary (Fig. 1). *KAN4/ATS* is expressed in the abaxial side of ii primordia and loss of *KAN4/ATS* function leads to the fusion of ii and oi into a single thicker integument [56]. KAN4/ATS is post-translationally repressed by the UNICORN (UCN) active AGC VIII kinase [19, 20]. In *ucn* mutants as in *sk21*-*D*, a *kan4/ats* dominant allele, integuments form protrusions that originate from periclinal or oblique divisions of dermal cells [19]. Moreover, KAN4/ATS interacts with DELLA proteins, repressors of gibberellin-dependent pathways, to regulate the expression of genes involved in integument growth [32]. In situ hybridization analyses of the class III homeodomain-leucine zipper transcription factors *PHB*, *PHV*, and *CNA* have revealed their ii adaxial pattern of expression [45, 75]. *phb;phv;cna* triple mutant displays a variety of phenotypes such as reduced ii or oi growth and the formation of amorphous integuments [45]. *PHB* expression in the ii is regulated by WUS, ANT, and SPLL/NZZ, whereas it is independent of INO and KAN4/ATS [45, 75]. Overall, these genetic analyses suggest that a balanced expression of adaxial and abaxial polarity determinants is necessary for both ii and oi growth, similar to what observed in leaves [45, 56].

#### Regulation of multiplicative integuments

The growth of Arabidopsis sub-dermal integument cell layers has been found to be tightly regulated. The development of ii sub-dermal cell layers (ii1′ and ii1″) by periclinal cell divisions of the ii1 is promoted by STK and TRANSPARENT TESTA 16 (TT16) MADS-Box transcription factors [13]. *tt16;stk* ovules lack ii sub-dermal cell layers almost completely. Furthermore, TT16 is a master regulator of ii1′ and ii1″ cell patterning [13, 15]. Wild-type ii1′ arrests before the micropylar region, thus creating a proximal–distal polarity in seed coat thickness. *tt16* ovules exhibit a distal shift of ii1′ and ii1″ development, thus creating a thicker micropylar and thinner chalazal region, compared to the wild type. *TT16* expression pattern in the ii1 marks in advance the development of the ii1′ and is responsible for the correct positioning and progression of ii1 periclinal cell divisions. In *tt16* seeds, embryo development is impaired by the mechanical action of the seed ii1′ invading the micropylar pole, highlighting the importance of tightly regulating sub-dermal integument growth. Finally, TT16 has been involved in the formation of oi sub-dermal cell stripes (oi′), which arise by periclinal cell divisions of sub-dermal chalazal cells [24]. The chalaza of *tt16* ovules appears more extended along the proximal distal axis, compared to the wild type, thus favoring the growth of sub-dermal cells in between oi1 and oi2.

After fertilization, ii1′ and ii1″ undergo dramatic cell expansion, compared to all other integument cells, thus affecting considerably seed coat thickness. The differentiation of all integument cell layers was thought to be repressed by FERTILIZATION INDEPENDENT SEED (FIS) Polycomb group (PcG) proteins (see below) and induced by a signaling pathway initiated by the endosperm [22, 70]. Nevertheless, genetic analyses indicate that ii1′ and ii1″ cell expansion is not repressed by FIS PcG proteins and requires a signal from both embryo and endosperm [13, 23]. ii1′ and ii1″ sub-dermal position is not responsible for such a unique regulatory mechanism as oi′ cell expansion responds to the same molecular pathways underlying the development of integument dermal cell layers [24].

#### Hormonal signaling

As described above, cytokinins and gibberellins regulate ovule proximal–distal [4] and adaxial/abaxial patterning [32], respectively. Brassinosteroids induce *ANT* and *HLL* while repressing *AP2* expression during early ovule development [37]. Finally, auxin has been shown to play a fundamental role in Arabidopsis integument initiation. Mutations in the auxin efflux facilitator PIN-FORMED 1 (PIN1) as well as treatments with the auxin flux inhibitor NPA lead to finger-like ovule structures [4]. SPL/NZZ and BEL1 regulate *PIN1* expression by acting through the cytokinin signaling pathway [4]. Furthermore, SPL/NZZ has a role in auxin homeostasis by repressing the expression of *YUCCA* genes, which are involved in the biosynthesis of auxin [51]. Likewise, NAC family transcription factors CUP-SHAPED COTYLEDON 1 (CUC1) and 2 promote integument formation by modulating auxin flux through a PIN1-dependent mechanism [42]. Auxin affects not only ovule proximal–distal, but also adaxial–abaxial patterning. KAN4/ATS interacts with ETTIN (ETT), also known as AUXIN RESPONSIVE FACTOR 3 (ARF3), on the abaxial side of the ii [46]. KAN4/ATS-ETT/ARF3 protein complex promotes ii laminar growth and oi–ii separation by regulating *PIN1* expression [46]. Finally, *microRNA167* has been shown to promote integument growth by preventing *ARF6* and *ARF8* expression in the ovule [91].

#### Kinase signaling

In addition to UCN (see above), a number of other Arabidopsis kinase proteins, acting in different signaling pathways, have been involved in ovule integument outgrowth.

The *ERECTA* (ER) gene family, encoding for LRR-receptor-like kinases (LRR-RLKs), is expressed in the ovule integuments and is important for proper integument growth [64]. When the expression level of *ER* and *ER*-*like* genes was dramatically reduced, the integuments failed to completely surround the nucellus. The *ER* gene family genetically interacts with the *PRETTY FEW SEEDS 2* (*PFS2*) homeo-box gene, which also promotes integument outgrowth [62, 64]. Similarly, mutations in the MITOGEN-ACTIVATED PROTEIN KINASES 3 (MPK3) and 6 (MPK6) lead to premature arrest of ii and oi growth and have been speculated to work downstream of the ERECTA pathway [86].

The LRR-RLK *strubbelig (sub)* mutant leads to incomplete and irregular growth of the oi [11]. SUB trafficking is mediated by HAPLESS13 (HAP13), the μ subunit of adaptor protein 1 that regulates protein sorting at the trans-Golgi network/early endosome. *hap13* ovules display reduced oi development probably due to SUB mistargeting [87]. A *sub*-like ovule phenotype was observed in *pfs2;ino* and *pfs2;nzz* ovules and was compared to integument lobes of primitive ovules [61]. A forward genetic approach, aimed at finding mutations causing *sub*-like ovule phenotypes, allowed Fulton and coworkers to identify the *quirky (qry), detorqueo* and *zerzaust* mutants [25]. *QKY* encodes a predicted membrane-anchored C2-domain protein that co-localizes with SUB at the plasmodesmata, thus raising the possibility that both proteins influence symplastic trafficking of molecules [84].

Finally, *ARABIDOPSIS CRINKLY 4* encodes another RLK protein localized at the plasma membrane of L1 cell layers [29]. In *acr4* mutants, ovule integuments display a wide range of phenotypes, including arrest of integument growth and lack of integument cell layer organization [29, 30, 89].

#### Epigenetic determinants

dsRNA processing and epigenetic control of transcriptional activation have been shown to regulate ovule integument development in Arabidopsis. Mutations in *SHORT INTEGUMENTS 1 (SIN1)* (also known as *SUSPENSOR1 or CARPEL FACTORY*), which encodes a Dicer protein, result in premature integument arrest [31]. Nevertheless, the RNA targets of SIN1 are yet to be identified.

As mentioned above, PcG proteins have also been involved in integument development. Two FIS components of the Polycomb repressive complex 2, MULTICOPY SUPPRESOR OF IRA1 (MSI1) and FERTILIZATION INDEPENDENT ENDOSPERM (FIE), act sporophytically to repress integument cell expansion and differentiation before fertilization [70]. The study of FIE and MSI1 is complicated by the seed lethal phenotype of loss of function mutants. Nevertheless, FIE and MSI1 are haploinsufficient and emasculated *fie/*+ and *msi1/*+ flowers produce both wild-type-looking ovules and enlarged autonomous seeds having a partially differentiated seed coat. Furthermore, Hennig and coworkers discovered a positive role for MSI1 in integument outgrowth [35]. *MSI1* co-suppression lines exhibited orthotropous ovules with limited oi development, resembling *ino* mutants.

## Conclusive remarks

**Fig. 3:**
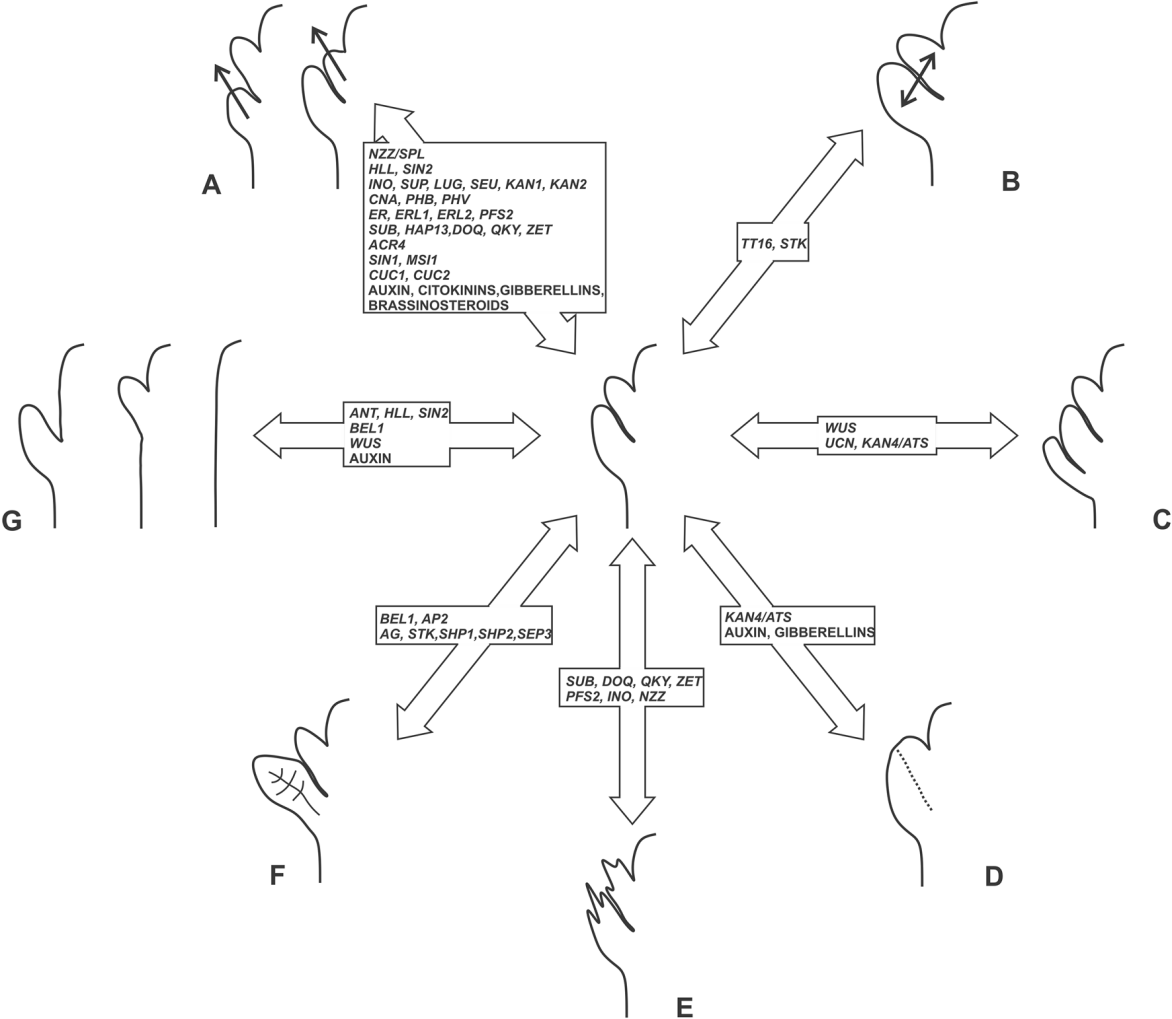
List of Arabidopsis genes and hormones known to regulate seed coat thickness

Genetic analyses of Arabidopsis ovule development revealed a number of master regulators of integument number and cell thickness that might well be responsible for the natural diversity observed in seed coat thickness (Fig. 3). Genes, such as *ANT*, *ATS*, *BEL1*, *INO*, and *ETT*, have been used as markers to better understand the evolution of unitegmy and ategmy in angiosperms [10, 53, 55]. Furthermore, the expression pattern of *AGL6*-*like* genes suggests that the angiosperm ii is homologous to the gymnosperm single integument [68]. By contrast, the evolution of the oi remains more controversial. It has been hypothesized that cupules might be ancestral ois. In line with this model, the Arabidopsis oi expresses *INO*, member of a gene family implicated in leaf development [85]. Moreover, Arabidopsis *ap2;bel1* and *stk;shp1;shp2;bel1* mutant ovules show a conversion of the oi into a leaf-like structure. Alternatively, the oi might have evolved de novo through the *WUS* or *UCN* pathway. Less attention has been given to arils while addressing the evolution of the oi. The study of gymnosperms B-sister MADS-box genes, orthologous to Arabidopsis *TT16* and *GORDITA*, revealed their high expression in the integument of *Ginkgo biloba* ovules and low expression in integument and aril of *T. baccata* ovules [54]. These data suggest, despite being interpreted differently by the authors of such study, that *T. baccata* arils express an integument molecular marker and might be seen as integuments. Expression analyses of other integument master regulator genes in angiosperms and gymnosperms arils would help better address nature and evolution of these integument-like structures.
